# A study on the correlation between seat selection and interaction preference in virtual-reality fusion simulation experiment

**DOI:** 10.3389/fpsyg.2022.1027959

**Published:** 2022-10-19

**Authors:** Shihan Chen, Yuan Luo, Hao Zhang, Xiaohong Liu

**Affiliations:** ^1^Office of Laboratory Administration, Xi’an University of Finance and Economics, Xi’an, China; ^2^Shaanxi Provincial Student Financial Aid Affairs Center, Education Department of Shaanxi Provincial Government, Xi’an, China; ^3^Graduate School, Shaanxi University of Chinese Medicine, Xianyang, China; ^4^School of Business, Xi’an University of Finance and Economics, Xi’an, China

**Keywords:** gamification, seat selection, interaction preference, virtual simulation, immersive teaching environment

## Abstract

In order to explore the correlation between students’ seat choice and interaction preference in the open gamification scenario, an experiment has been carried out on the platform of provincial virtual simulation experiment teaching center of a university, and tested the relationship between absolute distance, seat type, workstation type, and students’ interaction preference. The results show that in the virtual-reality fusion gamification scenario where students can move freely: (1) The inner circle students can stimulate the outer circle students’ willingness to invest in learning. (2) The task attribute and the seat distribution of the group may lead to the difference of students’ interaction preference. (3) Students are more likely to learn knowledge and skills by interacting with “people” rather than “object.” (4) Gender and major influence students’ experience of participating in gamified teaching. The results confirm that the interactive engagement effect of location does exist in immersive virtual-reality fusion gamification teaching scenario, and suggestions are put forward to adjust the effect of location through instructional design and teacher intervention.

## Introduction

Keeping students more motivated and participated in the classroom can potentially improve their learning effectiveness in teaching activities (Zhang et al., 2021). In recent years, more and more new instructional designs are emerging to stimulate student involvement. Among them, the concept of “gamification of teaching” is widely used in distance education and virtual simulation experiments. Gamification mainly refers to the use of game design elements in non-game contexts (Dymek, 2017), or the process of packaging teaching activities into game activities (Mishra, 2019). A number of studies on game motivation have proved that game elements can stimulate and strengthen learning motivation by creating challenges and fun, satisfying players’ basic psychological needs for relationship, ability and autonomy (Stansbury and Earnest, 2016). Based on this, the design of the simulated game environment can shift the student’s attention from mere memory to interpersonal communication and procedural cognition. The behavior in the scene realizes the overflow and complementation of group knowledge and improves the classroom effect through the communication and interaction between students and other subjects. Therefore, the interaction behavior of students in the simulation game environment is very different from that in the traditional teaching environment.

Although the theoretical framework of cognitive load theory has acknowledged a role for the learning environment (Paas and Merriënboer, 2020), the specific characteristics of the physical learning environment that could affect cognitive load have rarely been considered (Choi et al., 2014). Until 2014, the specific characteristics of the physical learning environment was verified can interact with learner characteristics, learning task characteristics, or the interaction terms of the two (Choi et al., 2014). For example, previous studies have shown that students’ perception of the scene was affected by their seating positions, and students in the high-interaction area have a higher rate of interaction. They show confidence, creativity and a strong sense of competition through rich social shared learning activities (Hong and Lee, 2017). These activities modulate the opportunities and levels of student engagement in the curriculum at different time scales and spatial contexts. However, few studies have focused on the impact of seat characteristics on students’ learning behaviors, such as the impact of different workstation designs and position orientations on students’ interaction intentions. In addition, most of the existing studies on seat preference and interaction in learning focus on the testing environment in small and medium-sized classroom spaces with a single seating type and layout type. Few scholars have discussed the change in learning interaction caused by students’ seat choice behavior in polymorphic layouts and more open learning environments, although this is an important part of the design of modern teaching environments (Lee et al., 2019).

In order to make up for the deficiencies in this research field, this study conducted an investigation in the laboratory of the provincial virtual simulation experiment teaching center of Economics and Management at Xi’an University of Finance and Economics. We discussed the relationship between student seat selection and learning interaction preferences in an immersive virtual-reality fusion gamified learning scenario. This study attempts to explain the indicators with significant differences between groups, and helps instructional designers and teachers to adjust teaching arrangements and implement teaching management in a targeted manner.

## Literature review and problem posing

### Gamified environment and classroom seating

Meaningful gamification forms an intrinsic motivational mechanism by promoting students to establish diverse connections with the real environment, cultivating students’ ability to think deeply and actively learn (Stansbury and Earnest, 2016). The combination of simulated teaching space design and virtual reality technology (VR) can effectively stimulate the senses and emotions of participants, enhance the reality experience, and provide course designers with a novel and challenging revolutionary teaching tool. In the immersive virtual-reality fusion learning scenario of virtual simulation experiment, the learning ecological environment is composed of the three-dimensional space of physical space, information space and virtual space (Yang and Zhang, 2021). The design of multimodal teaching gamification (Doumanis et al., 2019) needs to meet students’ requirements for immersion in three dimensions of space, emotion, and time (Jarvis, 2019).

The architectural space of the classroom directly affects the students’ course experience. And the visual perception of the classroom architectural space will deeply affect the students’ sense of belonging, willingness to discuss, the effect of information transmission, and the frequency and method of interaction (Chen, 2020). Architectural space includes all spatial features within the building such as space profile, decoration, light and circulation. The relative position of students in the building space can affect student performance, such as linear or walking distance from entrances, screens, aisles and the platform. Generally speaking, the seating arrangement in a classroom space determines where students are positioned relative to various space markers. Students’ perception of scenes is affected by their seating positions, that is, students’ seating positions and their learning outcomes may be correlated (Zomorodian et al., 2012; Seet et al., 2022). Seating preference, as a personality trait, may be affected by building space such as classroom area and resource allocation (Haghighi and Jusan, 2015). Meanwhile, seating preference has also been shown to be related to students’ self-concept such as proactive personality, characteristic, and personal space needs such as privacy preference (Gou et al., 2018). In traditional classrooms, the emotional, psychological and sociocultural environment provided by the active interaction area is superior. This superiority is mainly manifested in the closer relationship between learners, the more active learning atmosphere, the more efficient learners’ thinking mode, and the better learning habits. A good emotional psychological environment and sociocultural environment can shorten the emotional distance between learners, improve communication efficiency, and catalyze learners’ intrinsic cognitive processing (Li et al., 2018).

### Seating and learning interaction intention

Intensive class is the most adopted teaching method in university education, and the seats that students choose are almost in similar areas in different courses (Gou et al., 2018). Although the relationship between seating and student achievement is unclear, the impact of seating on students’ classroom performance and learning engagement is significant (Perkins and Wieman, 2005). Seat preference and seat choice reflect student personality traits related to student performance (Benedict and Hoag, 2014), such as cognition, subjective motivation, and expected engagement. There is ample evidence to support the positive impact of classroom “front row” and “center” locations on student engagement, either because students who want to participate in the classroom intentionally choose a location where they can interact with teachers and contents easily, or because teachers tend to pay more attention to students sitting in prominent positions in the classroom (Griesinger, 2018). Subsequent studies revised the conclusions about the positive interaction zone. Some scholars believe that although the outer corners of the front row are very close to the podium, the interaction between students and teachers in these positions is not high. The truly effective seating area is an equilateral or inverted triangle (Hemyari et al., 2012; Zomorodian et al., 2012; Liu et al., 2021). If the number of students asking questions is used as an observational indicator to measure students’ active engagement in learning, this active area will shrink further and show a T-shaped distribution (Marx et al., 1999).

Study reported that the purposeful arrangement of student seats at the beginning of the course can also arouse the enthusiasm of students. Even if they changed their seats halfway though, these students will not reduce their level of learning engagement by leaving the active interaction area of the classroom (Perkins and Wieman, 2005). Another study explained this phenomenon from the perspective of social roles, arguing that different seats represent different social identities (group roles). Students have the opportunity to accept or resist social identities (group roles) associated with specific seating positions. Once you try to accept a certain identity setting, this awareness will persist in a certain atmosphere and time (Parker et al., 2011). This suggests that seat selection and learning interaction willingness are correlated and likely to occur, rather than causal and predictable. In the simulated experimental environment, the group roles and job content played by students are preset and can be chosen independently. Seating preference predicts students’ recognition, acceptance, and performance confidence of the role and job content they choose.

### Seating and teaching intervention

Classroom physical space layout is an important medium of teaching and learning in modern education, which has a significant impact on curriculum design and teaching implementation. In the classroom space with wide space between seats, teachers pay more attention to individual students, teaching methods tend to be diversified, and teaching supervision is more effective (Kaya and Ağaoğlu, 2013). The study found that teaching activities and learning dominance almost depend on teachers when the seating arrangement in the classroom adopts the traditional teacher-student face-to-face, single-person and single-table form (vertical distribution). However, when the seating arrangement in the classroom adopts the form of innovative scattered distribution, multi-person circle (horizontal distribution), the teaching activities and learning dominance mainly depend on the students. In the process of the seating arrangement from vertical distribution to horizontal distribution, the situation that students are marked differently because of the distance from teachers is significantly reduced, and the focus of curriculum assessment is gradually changed from learning results to learning process (Doménech Betoret and Gómez Artiga, 2004). In addition, vertically distributed spaces are more suitable for courses that require comprehension and memorization, while horizontally distributed spaces are more effective when applied to analytical and cooperative learning activities (Doménech Betoret and Gómez Artiga, 2004).

Horizontally distributed classroom space can effectively promote student interaction. When students can decide their physical location in the learning environment by themselves, the interaction quality will significantly improve (Rae and Sands, 2013), and the learning efficiency and attitude are also maintained at a high level (Shi and Wang, 2018). In response to this informal classroom, instructional designers should make adaptive changes in the structure of the curriculum, to encourage students’ spontaneous behavior and free interchange of idea. Trial and error should also be encouraged, leading students to ask questions, express ideas, negotiate, express grievances, ask for explanations, and accept the viewpoints of others (Said et al., 2015). All in all, different classroom physical space layouts need to match different teaching orientations, teaching methods, learning task settings, assessment requirements and even teaching workloads in order to play a better seat position effect.

This study rediscusses the relationship between seat choice and interactive behavior in learning in the context of simulation game teaching, especially considering the influence of seat type. In combination with previous research, it aims to clarify the following questions:

1. How does the interaction level between students and different subjects in the immersive virtual-reality fusion gamified learning scenario?
2. Whether the preference and degree of interaction is related to seat selection?
3. Whether this interactive preference difference can be moderated?

## Materials and methods

### Research context

This work relies on the platform of the provincial virtual simulation experiment teaching center of economics and management at Xi’an University of Finance and Economics in China to carry out related research. The center’s laboratory covers an area of 1,000 square meters and has five simulated parts: business service area, financial service area, government service area, manufacturing park and trade park. It can accommodate nearly 300 students to carry out virtual simulation experiments at the same time, and undertake interdisciplinary comprehensive experimental teaching tasks for about 3,000 students from the school of economics, school of management and school of business every year. The construction of the laboratory uses a number of modern educational technologies such as virtual reality, multimedia, human-computer interaction, database and network communication, aiming to provide an integrated, immersive and interactive learning environment for students.

This study is implemented in parallel with the regular interdisciplinary comprehensive practical training courses of economics and management conducted by the university. The interdisciplinary comprehensive practical training courses of economics and management is a comprehensive collaborative confrontation experiment that simulates the realization of business interactions between manufacturing enterprises and external service organizations such as Municipal Supervision Bureau, Taxation Bureau, banks, and domestic logistics companies through an information technology platform under certain data models and business rules, in order to help students experience the whole process of establishing and producing, operating and managing of an enterprise.

So far, the course has been conducted for 44 sessions in the past 5 years, and we have followed the whole teaching process after the 18th session and participated in the revision of the successive teaching plans. Based on previous experience in teaching organization and the requirements of the established experimental objectives, the teaching team has developed a detailed teaching program to ensure that same teaching strategies and game plans are used in each round of the experiment, with controlled pacing and strict implementation. We present a condensed version of this program in Table 1.

**Table 1:** Teaching schedule (condensed version).

Date	Student action	Teacher action
Day 1 Morning	Mobilization conference	Course basic information
	CEO campaign	CEO registration
		CEO competitive speech
Day 1 Afternoon	Recruitment conference:	Resume guidance
	Fill out the application registration form for an interview	Career planning guidance
	Team formation, team building	
	Team appearance	
Day 1 Evening	Individual registration	Training in writing business plans and creating Road Show PPTs
	Create a roster of employees	Explanation of business registration process
	Learn business registration process and registration form filling specifications	Pre-job training for peripheral institution personnel
	Peripheral institutions make registration form templates	Pre-job training for enterprise finance personnel
	Design company logo, draw corporate posters, etc.	
	Prepare PPT for Road Show and complete personal work log	
Day 2 Morning	Familiar with operation rules	Teachers explain the business operation specifications, knowledge points involved and precautions for each institution
		Explain the process of loan and tax payment
	Company registration	Guiding students through the registration process
		Conducting the excellent poster competition
Day 2 Afternoon	Trial operation for 4 quarters, familiar with software operation and operation rules	Answer questions from students
		End of the trial operation, CEO regular meeting
Day 2 Evening	Make business plan and Road Show PPT	
	Summarize work plan, business process and form a copy	
Day 3	Official operation	Answer questions from students and tutor experiments
Day 4	Official operation	Answer questions from students and tutor experiments
	Road Show	Road Show
Day 5 Morning	Official operation	Answer questions from students and tutor experiments
	Summary	Statistical operating results
	Organize course materials	
Day 5 Afternoon	Summary conference:	Preside over the meeting
	Team representatives take the stage to summarize	Announcement of results
	Submission of course materials	

It is worth emphasizing that, unlike the role of teachers in the traditional classroom, the teaching team developed a semi-autonomous teaching mechanism adapted to the open environment, encouraging full and free action of students. Due to the specificity of the experimental course content, the teachers apply more role-playing, task-driven and case-based teaching methods for teaching organization throughout the experimental process. Four instructors, which must include one with a background in economics discipline, one in computer discipline, one in business discipline and one in management discipline, can help students from different majors to solve experimental technical problems and business processing problems, and reduce the risk of redundant student interactions in the experiment.

**Figure 1:**
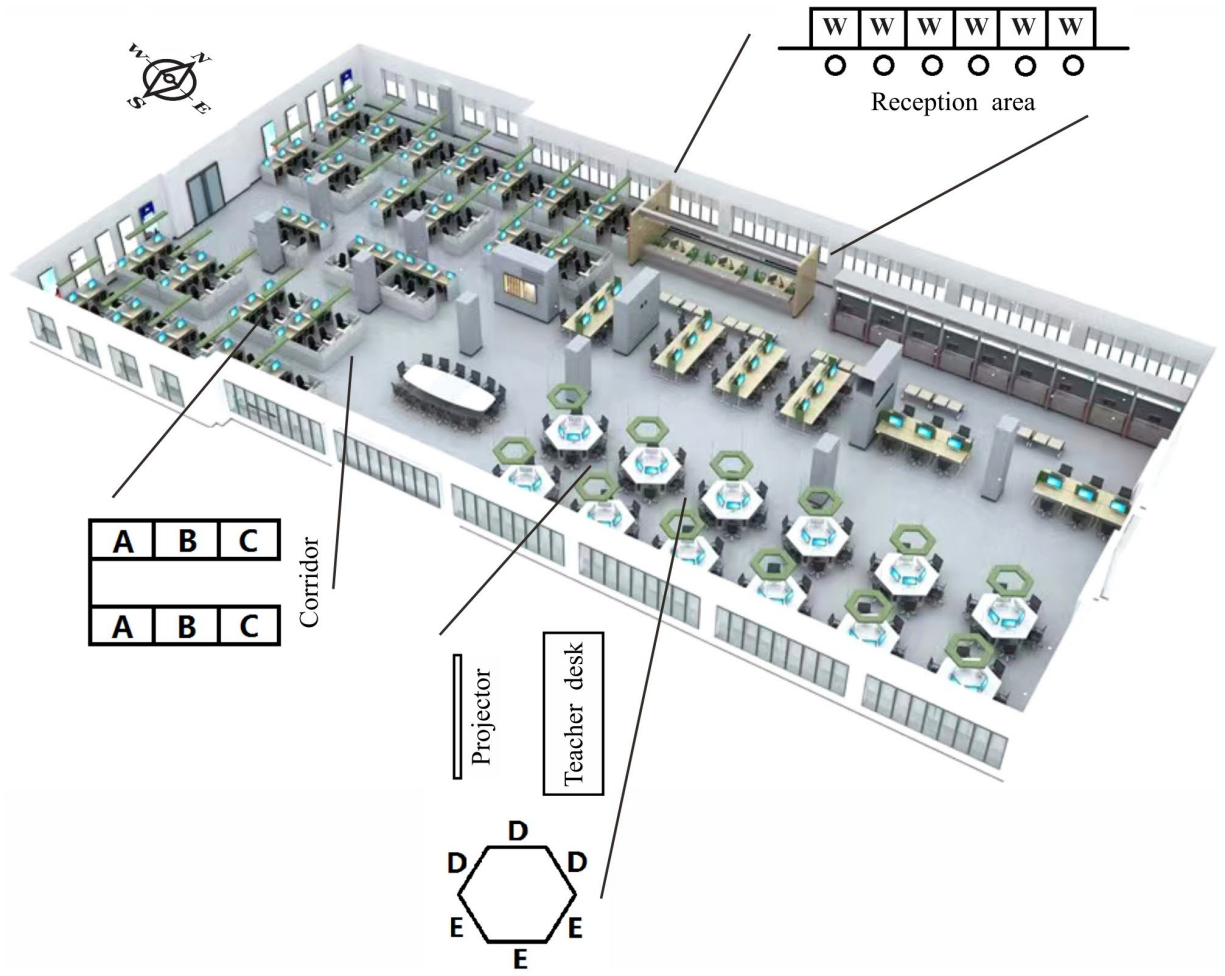
Layout of the classroom and seat code.

Figure 1 shows the layout of the classroom and describes three main workstation types in a schematic diagram, which are classified and coded according to the spatial concealment of different seating types. The manufacturing park is distributed with all the simulated manufacturing companies (west side of the classroom). The workstations in the manufacturing park are semi-enclosed cubicle, in which seat A is close to the partition, seat C is close to the corridor, and seat B is in the middle. The trade park is distributed with all simulated suppliers and traders (southeast side of the classroom). The workstations in the trade park are open round tables, in which seat D backs to the central conference table, so teachers can clearly see students’ behaviors sitting here. Seat E faces the central conference table, but there is a blind field of vision, so teachers cannot directly observe the students in this position. The financial and government service area has a window-type service desk with no difference for each seat (encode W), where located the offices of banks, Municipal Supervision Bureau and Taxation Bureau (northeast of the classroom).

### Instrument (5-D student interaction scale)

#### Online self-regulation questionnaire

Refer to the Online Self-regulation Questionnaire (OSRQ) developed by Cho and Cho (2017) to describe three types of interaction in online learning environment (student–content interaction/student–student interaction/student–teacher interaction), which tend to measure the level of interaction between students and content, classmates and teachers in the course. The topic involves four aspects: initiative of interaction, acceptance of interaction, interaction skills, and interaction effect. Items of the questionnaire have been appropriately adjusted according to the experimental situation, and the specific elements involved in the course are integrated with the elaboration of the topic. Options used a Likert 7-level scale.

#### Student–environment interaction efficacy scale

The course provides students with a simulated gamification experience by simulating the organization, rules and laws of a real business social environment. Based on the interface logic of the online interdisciplinary comprehensive training platform of Fangyu, a Student–Interface Interaction Scale is designed to evaluate students’ familiarity with each functional module and the intensity of operational investment. In addition, we designed a Student–Space Interaction Scale to evaluate whether students have engaged in self-directed learning through active spatial exploration behaviors. Table 2 shows that all the scales above have passed the reliability analysis.

**Table 2:** Descriptive statistics, reliability, and validity tests of scale dimensions.

Dimension	Number of questions	Mean	Standard deviation	α coefficient	CR	AVE
Student–content interaction	11	5.998	0.946	0.975	0.978	0.803
Student–teacher interaction	9	5.700	1.199	0.949	0.961	0.732
Student–student interaction	10	5.931	1.044	0.959	0.968	0.752
Student–space interaction	8	5.467	1.417	0.884	0.909	0.557
Student–interface interaction	5	5.474	1.159	0.867	0.906	0.660
Total interaction	—	5.714	1.166	—	—	—

### Research process

**Figure 2:**
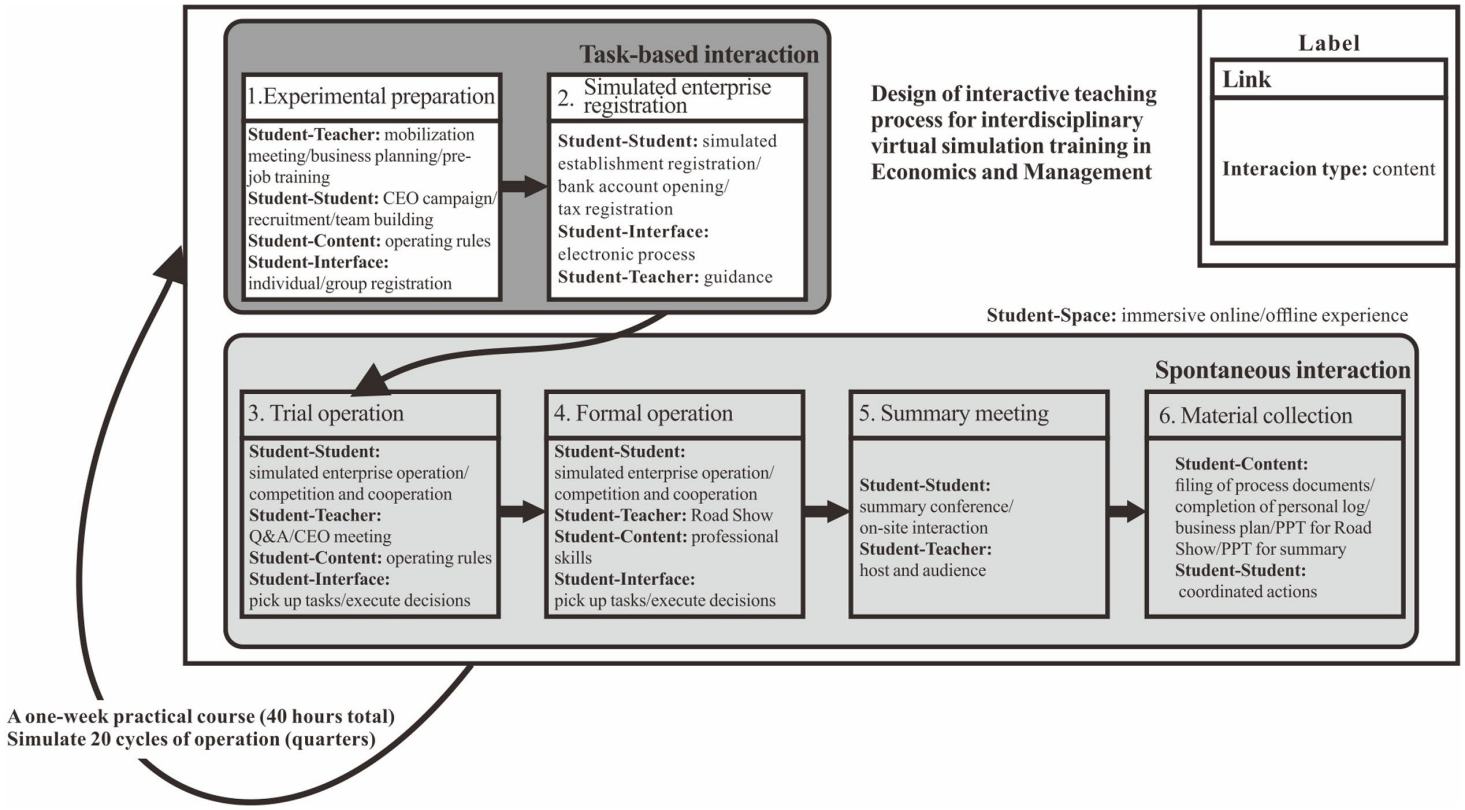
Design of interactive teaching process for interdisciplinary virtual simulation training in economics and management.

A study was conducted on the correlation between seat choice and interaction preference of students who participated in the 43rd and 44th interdisciplinary comprehensive practical training courses of economics and management in 2021. We were involved in the teaching of the 43rd and 44th courses and confirmed that both experiments were carried out strictly according to the teaching schedule developed. Before the study, we sorted out the design diagram of teaching interaction process according to the teaching plan of the latest version of the course. Figure 2 shows all teaching links and interaction contents among different interaction subjects. In the experimental preparation stage and the simulated enterprise registration stage, students are guided to interact mainly by setting predetermined tasks, and task-based interaction behaviors mainly occur in these stages. During the trial operation, formal operation, summary meeting and material collection stage, it requires all team members to discuss, make decisions, plan and execute on their own, and spontaneous interaction behaviors mainly occur in these stages. As can be visualized in Figure 2, during the whole training process, five types of interactions are evenly distributed and persist, thus research samples can fully experience different types of interaction and ensure the validity of data.

The core simulation organization sets up CEO, financial manager, purchasing manager, production manager, sales manager, marketing manager and other simulated functional positions. The training requires that each group consists of at least 4 students from different majors, and at most 2 people in the same major are in order to achieve the purpose of reasonable division of labor, corresponding positions and majors, and effective teamwork (Due to the highly specialized work content of some institutions, this study does not require inter-professional arrangements such as the Municipal Supervision Bureau, the Taxation Bureau, Banks, and Media Companies). Therefore, students have a lot of freedom in selecting groups, positions and seats in the on-site recruitment process, and their preference for positions can be fully reflected in the results of entering the group.

## Data collection and analysis

### Statistics and testing

In this study, questionnaires were distributed to all students who participated in the 43rd and 44th experiments through the Questionnaire Star platform. A total of 402 questionnaires were distributed and 345 were recovered, with a recovery rate of 85.82%. After removing the unqualified questionnaires due to invalid samples and incorrect confirmatory items, 330 valid questionnaires were obtained, with an effective rate of 95.65%. Using SPSS 18.0 and Excel tools to test the reliability and validity of the questionnaire, the results are shown in Table 2. In all dimensions, the minimum value of Cronbach’s alpha coefficient was 0.867, and the minimum value of CR was 0.906, which were all higher than the critical value of 0.7, indicating that the reliability of the questionnaire was good. All factor loadings were greater than the critical value of 0.5 (0.623–0.943) at the level of *p* < 0.001; the average variance extraction (AVE) of the dimensions ranged from 0.557 to 0.803, all of which were greater than the critical value of 0.5, indicating that the convergent validity of the questionnaire was ideal.

### Distribution of seat selection

Statistics show that of the total 330 valid samples, 73 people chose seat A, 75 chose seat B, 79 chose seat C, 26 chose seat D, 49 chose seat E, and 28 chose seat W. It shows the selection of seats A, B, and C had the largest and equal number of sample feedbacks, indicating that the samples participating in the survey are mainly from simulated manufacturing enterprises, and the seats selected are evenly distributed. At the round table workstations, more people chose E-shaped seats than D-shaped ones (the round table workstation can seat 6 students, but the course only arranged 4–5 students according to the actual setting of the institution, so students have a higher option to drop E or D). The above findings suggest that many students tend to avoid teachers’ supervision, rather than welcome teachers to guide them at any time.

### Interaction level analysis

The descriptive statistics of each dimension of the 5-D Student Interaction Scale are shown in Table 2. In general, the level of interaction between students and learning content such as operating rules and vocational skills is the highest (5.998). Then the interaction levels of students are ranked as student–student (5.931), student–teacher (5.700), student–interface (5.474), student–space (5.467). It means that knowledge learning is still the main goal for students to participate in the classroom in a simulation game-based teaching, and communication effect between people is better than that between people and the environment. After averaging the five dimensions with equal weights, the average of overall interaction level of the samples is 5.714, which is between the self-efficacy ratings of “relatively consistent with some kind of positive interaction” and “very consistent with some kind of positive interaction.” The above results show that the students participating in the interdisciplinary comprehensive practical training courses of economics and management have better overall interaction and stronger self-investment.

**Figure 3:**
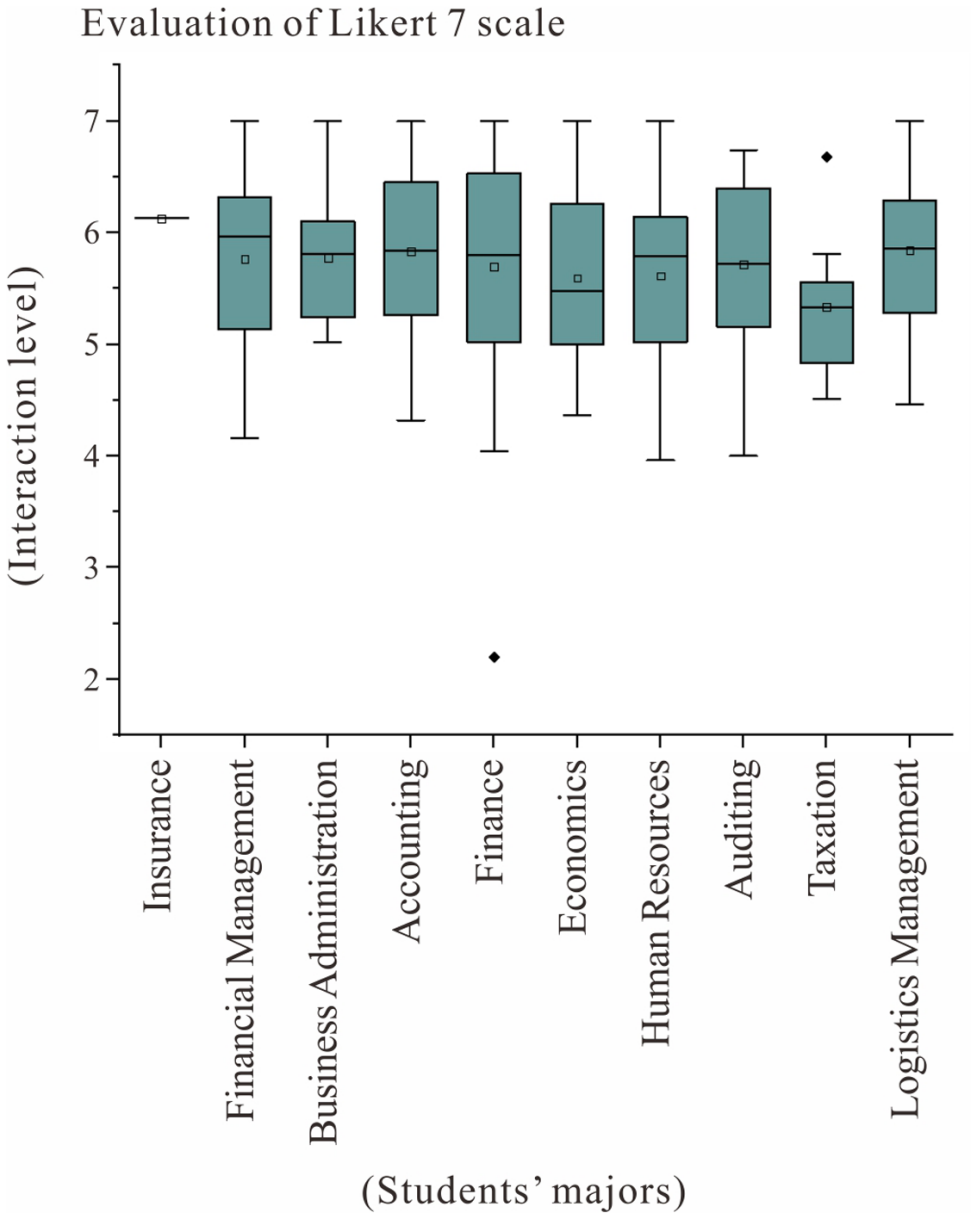
Box plots of the total interaction levels of students in different majors in the experiment.

Figure 3 shows the boxplots of the overall interaction levels of students in different majors. As shown in the figure, students majoring in Taxation showed relatively low interactive satisfaction, students majoring in Finance had the most discrete self-rating distribution, and only one person majoring in Insurance participated in the survey, which resulting in invalid statistical results. All Business Administration students gave a self-efficacy rating higher than “relatively consistent with some kind of positive interaction.” Therefore, the preliminary observations of this study show that there are differences in the depth of communication between students of different majors in the simulation experiment.

## Results

This work examines the relationship between student interaction preference and seat type, workstation type, as well as absolute distance between workstation center point and teachers’ desk center point, respectively. In addition, the influence of gender and major differences on students’ interaction preferences are found.

### The relationship between absolute distance and student interaction preference

**Figure 4:**
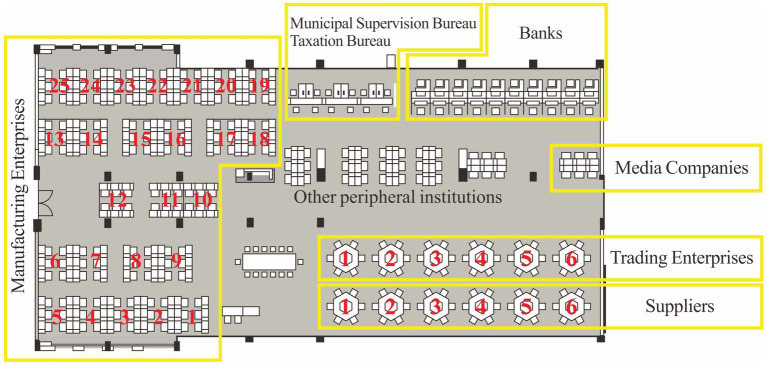
Classroom floor plan, main functional areas, and workstation codes.

**Figure 5:**
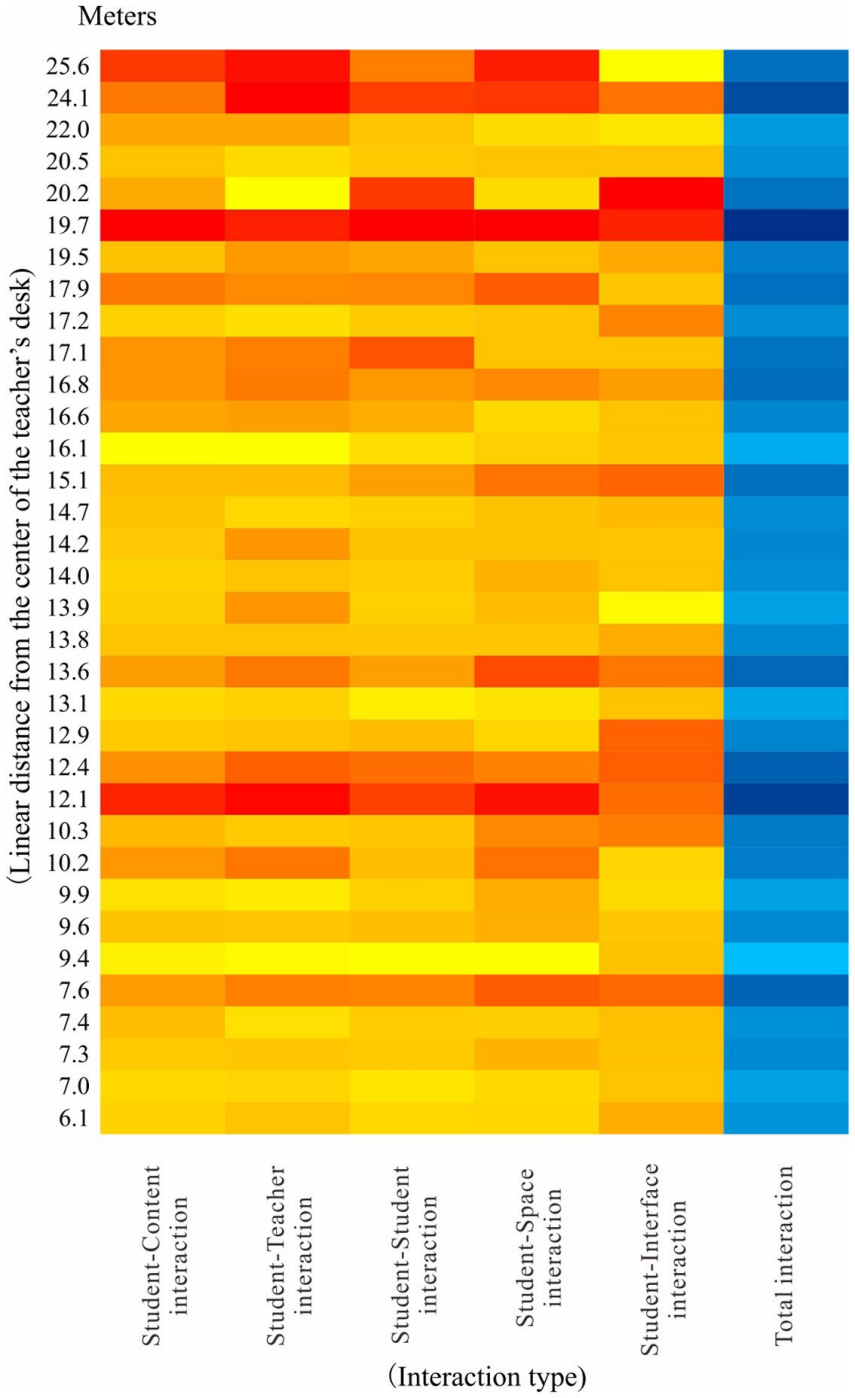
Heat map of student interaction preferences at different absolute distances.

**Figure 6:**
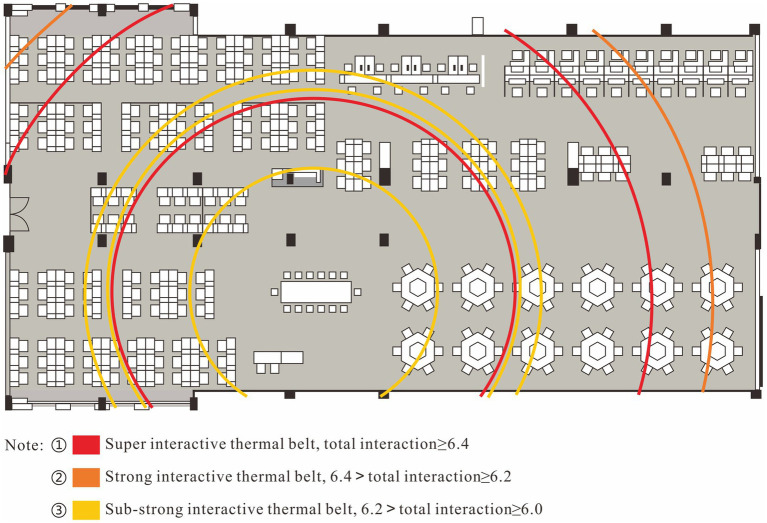
Spatial display of the interactive thermal belt.

The workstation codes of the simulated manufacturing enterprise, simulated supply enterprise and simulated trading enterprise are shown in Figure 4. In order to verify the possible impact of geographical differences, this study measured the straight-line distance between the center point of each institution’s workstation and the center point of the teachers’ desk (central conference table), combined with the level of student reported self-interaction at different workstations, drew an absolute distance-interaction preference heat map (Figure 5). The heat map shows that there are two super interactive thermal belts at 12.1 and 19.7 m from the center point of the teachers’ desk. There is a strong interactive thermal belt 24.1 meters away from the center of the teachers’ desk. The sub-strong interactive thermal belts appear at 7.6, 12.4, and 13.6 m away from the center of the teachers’ desk. We plotted the locations of these interacting thermal belts on the classroom floor plan at equal scale (Figure 6) to facilitate visualization of this result.

We counted the operation results of the simulation experiments in phase 43rd and 44th, and found that there are 2 (phase 43rd) and 3 (phase 44th) groups, respectively, in the top three operating performance (ranked by owners’ equity) are located on the thermal belts we discovered, including manufacturing enterprise 1, manufacturing enterprise 3, manufacturing enterprise 7, supplier 1, and trading enterprise 3 (refer to Figure 4 for the workstation codes shown). We believe that prominent learning effects may be more likely to occur in areas where interactions are active.

### The relationship between seat type and student interaction preference

*T*-test results (Table 3) show that there was a significant difference in the total interaction level between students who chose seat A or B in the semi-enclosed cubicle and students who chose seat C. When the student chooses the seat C next to the corridor, he or she will invest more in the study of experimental rules, office skills and vocational skills, and will prefer to communicate with other students, maybe has a stronger desire to explore the online interface environment or the space environment he stays. Although grid design does not affect the frequency and quality of interaction between students and teachers in a whole group, students in A or B seats still show a lower level of student–teacher interaction than other classmates working at the window-type service desk. Since the work content of “employees” in peripheral institutions is not closely related to the production, operation and management activities of the simulated enterprises, some students who choose W seat do not have a systematic and comprehensive understanding of the simulation software Fangyu, which directly leads to the fact that the total interaction level of students in peripheral institutions is significantly lower than that of students in seat C. Table 3 reports that students who choose W seats have significantly lower levels of interaction with the interface. In addition, this study found no significant difference between the seat choice and interaction preference of students who chose seat D or seat E. This shows that the open round table can bring them a more balanced and relatively stable interactive experience.

**Table 3:** Relationship between seat type and interaction preference.

Independent variable (seat)	*N*	Student–content	Student–teacher	Student–student	Student–space	Student–interface	Total interaction
A/B type seat	148	5.863	5.584	5.835	5.364	5.497	5.629
C type seat	79	6.156	5.769	6.071	5.658	5.775	5.885
Sig. (two-sided)	—	0.018^**^	0.177	0.056^*^	0.042^**^	0.011^**^	0.017^**^
A/B type seat	148	5.863	5.584	5.835	5.364	5.497	5.629
W type seat	28	6.127	6.004	5.854	5.452	4.121	5.511
Sig. (two-sided)	—	0.161	0.045^**^	0.922	0.690	0.000^***^	0.473
C type seat	79	6.156	5.769	6.071	5.658	5.775	5.885
W type seat	28	6.127	6.004	5.854	5.452	4.121	5.511
Sig. (two-sided)	—	0.873	0.258	0.278	0.360	0.000^***^	0.025^**^
D type seat	26	6.147	5.834	6.085	5.434	5.531	5.806
E type seat	49	6.002	5.699	5.955	5.507	5.661	5.765
Sig. (two-sided)	—	0.409	0.547	0.518	0.782	0.475	0.816

* *p* < 0.1;

** *p* < 0.05;

*** *p* < 0.01.

This study also analyzed the relationship between workstation type and student interaction preference, but found no valid results, which shows that the semi-enclosed cubicles, open round tables and window-type service desks bring students a diverse learning and office environment experience, but will not affect the overall learning input of the group.

### Further discussion of interaction preferences

The study found that gender affects how students interact with learning environment. In fact, boys tend to show a stronger willingness to communicate. They prefer to exploit the market, conduct cooperative consultations, build alliances or lobbying and gaming, rather than just sitting in their seats and studying rules and strategies. The operational results of previous trainings show that although the number of female students in Xi’an University of Finance and Economics far exceeds that of male students, the group leaders (simulated CEOs) who have achieved outstanding results were more likely to be male. Table 4 shows that boys are significantly more familiar with classroom space and operation interface than girls, and their total interaction level is 0.208 units higher than girls.

**Table 4:** Relationship between sample characteristics and interaction preference.

Independent variable (gender)	*N*	Student–content	Student–teacher	Student–student	Student–space	Student–interface	Total interaction
Male	80	6.023	5.846	6.071	5.717	5.705	5.872
Female	250	5.991	5.654	5.886	5.389	5.400	5.664
Sig. (two-sided)	—	0.767	0.128	0.102	0.021^**^	0.010^***^	0.034^**^
**Independent variable (major)**							
Accounting	57	6.171	5.948	6.093	5.614	5.344	5.834
Economics	38	5.820	5.550	5.771	5.317	5.505	5.593
Sig. (two-sided)	—	0.050^**^	0.042^**^	0.078^*^	0.151	0.413	0.128
Accounting	57	6.171	5.948	6.093	5.614	5.344	5.834
Human resources	49	5.907	5.496	5.792	5.320	5.555	5.614
Sig. (two-sided)	—	0.092^*^	0.014^**^	0.079^*^	0.141	0.200	0.133
Financial management	34	5.992	5.814	5.900	5.509	5.618	5.767
Auditing	31	6.138	5.731	6.013	5.485	5.207	5.715
Sig. (two-sided)	—	0.469	0.727	0.605	0.932	0.089^*^	0.799
Logistics management	32	6.074	5.778	6.063	5.623	5.669	5.841
Auditing	31	6.138	5.731	6.013	5.485	5.207	5.715
Sig. (two-sided)	—	0.735	0.845	0.803	0.617	0.064^*^	0.474

* *p* < 0.1;

** *p* < 0.05;

*** *p* < 0.01.

Major differences also lead to differences in students’ interactive preferences. Table 4 shows that compared with students majoring in Economics and Human Resources, students majoring in Accounting perform better in student–content interaction, student–teacher interaction, and student–student interaction. Compared with students majoring in Financial Management and Logistics Management, students majoring in Auditing have poor interface interaction performance. We tried to explain this phenomenon from the perspectives of subject classification, simulated job preferences, learning styles, etc., but no valid evidence was obtained.

## Discussion

This study confirms that in the immersive virtual-reality fusion gamification scenario, seat selection is related to the level of interaction between students and different subjects, and the location effect does exist. In a polymorphic layout and more open learning environment, there are strong interactive thermal belts and weak interactive zones both in the inner seat (front row or middle position) and outer seat (rear row). When there are concealment differences in the seat design of student workstations, the ones who seat in the hidden areas are more likely to feel slack and may reduce their willingness to communicate. Previous studies have shown that students’ participation in gamified classrooms depends on their attitudes toward games and their acceptance of teaching reform or dynamic (Byl and Hooper, 2013). In this study, we argue that the position effect can be moderated by instructional design and teacher intervention(Grzegorczyk, 2019). Based on this viewpoint, we propose discussions after analyzing the data.

### Prominent learning effects are more likely to occur in the inner circle area where the interaction is active: Students in the inner circle can stimulate the willingness of students in the outer circle to learn in the virtual-reality fusion gamification scenario where students can move freely

Some studies have found that the autonomous motivation of the students in smart classroom is significantly related to their seat preference whether or not close to the podium (Liu et al., 2021). But this conclusion is complemented by the two long-range active interaction bands shown in Figure 6. In the gamified scenario where students can move freely, students’ autonomous motivation was not significantly related to seat preference near or far from the podium. We believe that students in the inner circle with strong motivation have a catfish effect in the scene, which activates the willingness of students in the outer circle to engage in learning. In other words, when the inner circle students with strong motivation are given absolute freedom of interaction, they will establish a certain identity relationship with the outer circle students. Once the outer circle students identify with the identity, their learning engagement will remain at a high level. This means that under the premise of sufficient classroom space (Parker et al., 2011), teachers can promote the interaction of students in the inner circle (front row) and the outer circle (back row) by means of centralized seminars, regular centralized meetings, and long-distance cross-group cooperation. Teachers can also encourage dialectics and games among students, guide them to share multiple viewpoints to create divergent thinking methods. If the course requires teamwork, teachers can reversely adjust the order of workstations according to the situation of self-construction of students in the early stage, and arrange those students with weak motivation to study in the strong interaction area.

### Workstation design will not affect the overall learning engagement level of the group, but the task attributes of the group and the seat distribution of the workstations will lead to differences in students’ interaction preferences

This study verifies that different design of student workstations in the simulation experiment classroom will not affect the overall learning input level of a group, such as semi-enclosed cubicles, open round tables and window-type service desks. This conclusion validates the effectiveness of group collaborative learning in improving student engagement. In most cases, students’ acceptance or rejection of the group role represented by a position is largely determined by the subconscious rather than conscious and rational (Parker et al., 2011), means that students’ roles in the classroom and cognitive motivations can be manipulated. When the classroom is equipped with semi-enclosed cubicles (or a position right next to the wall), teachers need to focus on those students in the inner seats. At the same time, teachers should emphasize the importance of learning roles, encourage them to participate in long-distance cross-group collaboration, and incorporate this content into the design of teaching links and the scope of student assessment. If students need to complete their learning tasks through role-playing, they can ask the group to establish a job rotation system in order to select the most suitable position. For groups undertaking special learning tasks, instructional designers need to enrich and refine the work content of these special positions, enhance the group interactive experiences of virtual-reality fusion environment, and set up special assessment systems and evaluation standards.

### The main purpose of students’ participation in the simulation gamification course is to learn knowledge and skills, and they expect to achieve this goal through interaction with “people” rather than “object”

Current research often takes students’ motivation, willingness and emotional experience as learning outcome evaluation indicators in gamified environments, and few studies evaluated students’ engagement in learning contents or changes in learning beliefs (Stansbury and Earnest, 2016). This study demonstrates that students’ engagement in learning contents almost achieves a “very positive interaction effect” in a simulated gamification scenario. We found that mutual consultation among classmates is the preferred way for students to learn knowledge and skills in an open learning environment, including active observation, peer tutoring, mutual collaboration and other forms. In addition, students prefer to take the initiative to seek help from teachers when they encounter problems, rather than being checked and asked by teachers at any time when there are no problems. It is recommended that teachers should leave enough space for students and supervise rather than monitor their learning progress, behaviors such as verbal proximity, eye contact, or non-verbal cues can be used to maintain students’ attention. It should be emphasized that environmental elements also play an important role, including space exploration, reasonable peripheral participation, game element design, etc. Teachers can combine the characteristics of the classroom environment and use more innovative, interactive and conductive teaching methods, so that students can learn as efficiently as in the traditional classroom and get a better learning experience.

### Gender and major influence students’ experience of participating in gamified teaching

Firstly, boys tend to show a stronger willingness to communicate, and their interest in exploring virtual and real environments is significantly higher than that of girls. Secondly, compared with non-specialized courses, when students participate in specialized courses, their seating preferences are more strongly correlated with academic performance (Kalinowski and Taper, 2007), and there may also be differences in interaction habits. Therefore, it is necessary to advocate teaching students in accordance with their aptitude, deal with the academic problems of students of different genders and majors in a differentiated way, and encourage them to thorough think from specialized perspective.

Although gender and major are constant variables that cannot be changed, we still report this result because it can tell us whether we need to use location effects to moderate elements that are not conducive to interaction. Previous research has shown that a person’s personality traits, emotions, and behavior patterns can be influenced by physical environment. When people do the same thing in different background, they may have different outcomes due to contextual differences (Shi and Wang, 2018). We believe that the relationship between student behavior and the physical space distribution of a classroom is intricate, because learning effect is the result of a joint action of factors such as teaching style, learning ability and learning atmosphere. As stated in the Reciprocal Determinism proposed by Bandura (Yin et al., 2021), individuals, behaviors and environments are in a continuous interaction, the change of any one of these elements will cause mutual impact between any two of them. That is, students’ learning behavior is jointly regulated by multiple subjects such as teachers, classmates, and space, thus the position effect needs to be combined with other variables to achieve the directional action.

## Conclusion

This study discusses the relationship between students’ seating choices (especially considering the effect of seating type) and learning interaction preferences in an immersive virtual-reality fusion gamified learning scenario. The results show that seating choice and the level of interaction between students and different subjects are related and that location effects do exist. Students’ interaction preferences were particularly influenced by the absolute distance from the instructor’s location and the specific seat distribution, with non-variables such as gender and major possibly playing a mediating role and variables such as learning task attributes and teaching strategies playing a moderating role. The research design breaks through the limitations of the experimental environment selection for internationally related topics, and the findings demonstrate that immersive virtual-reality fusion gamified learning scenario foster different learner competencies than traditional learning scenarios (Garay-Rondero et al., 2019). We believe that it is an inevitable trend to further develop the comparative study of learner behavior characteristics in open environments, because the design of modern teaching environments will give new connotative characteristics to learning behavior.

Future research can combine algorithmic analysis to identify and model the trajectories of students’ interaction actions in the field, expand the sample size to verify the association between seat preference, seat type, seat matching and learning interaction preferences with it is engagement levels in an open environment, customize smart strategies to improve students’ learning experience and enhance students’ motivation to learn. In order to build a student-centered and technology-enabled ecological learning environment, teaching in an open environment needs to pay more attention to learners’ subjective characteristics, stimulate learners’ autonomous motivation, and expand the strong interactive area of an open space.

## Data availability statement

The raw data supporting the conclusions of this article will be made available by the authors, without undue reservation.

## Ethics statement

The studies involving human participants were reviewed and approved by IEC of The Affiliated Hospital of Shaanxi University of Traditional Chinese Medicine. The patients/participants provided their written informed consent to participate in this study.

## Author contributions

SC implemented this study and was responsible for data collection and analysis and writing. YL and HZ supported the investigation and data analysis. XL provided assistance in reviewing the manuscript. All authors contributed to the article and approved the submitted version.

## Funding

This research was funded by the Shaanxi Higher Education Teaching Reform Research Project with funding number: 19BY096, and by the Shaanxi Provincial Education Scientific Planning Leading Group Office with the funding number: SGH20Y1178 to SC.

## Conflict of interest

The authors declare that the research was conducted in the absence of any commercial or financial relationships that could be construed as a potential conflict of interest.

## Publisher’s note

All claims expressed in this article are solely those of the authors and do not necessarily represent those of their affiliated organizations, or those of the publisher, the editors and the reviewers. Any product that may be evaluated in this article, or claim that may be made by its manufacturer, is not guaranteed or endorsed by the publisher.

## References

[ref1] Benedict M. E. Hoag J. (2014). Seating location in large lectures: are seating preferences or location related to course performance? J. Econ. Educ. 35, 215–231. doi: 10.3200/JECE.35.3.215-231

[ref001] Byl P.d. Hooper J. (2013). “Key Attributes of Engagement in a Gamified Learning Environment”, in 30th ascilite Conference 2013 Proceedings. eds. Carter H. Gosper M. Hedberg J. (Sydney: Electric Dreams), 221–230.

[ref2] Chen M. (2020). A-STEM learning space design from the perspective of embodied cognition theory. Glob. Educ. 49, 46–57.

[ref3] Cho M.-H. Cho Y. (2017). Self-regulation in three types of online interaction: a scale development. Distance Educ. 38, 70–83. doi: 10.1080/01587919.2017.1299563

[ref4] Choi H.-H. van Merriënboer J. J. G. Paas F. (2014). Effects of the physical environment on cognitive load and learning: towards a new model of cognitive load. Educ. Psychol. Rev. 26, 225–244. doi: 10.1007/s10648-014-9262-6

[ref5] Doménech Betoret F. Gómez Artiga A. (2004). Trainee teachers’ conceptions of teaching and learning, classroom layout and exam design. Educ. Stud. 30, 355–372. doi: 10.1080/0305569042000310309

[ref6] Doumanis I. Economou D. Sim G. R. Porter S. (2019). The impact of multimodal collaborative virtual environments on learning: a gamified online debate. Comput. Educ. 130, 121–138. doi: 10.1016/j.compedu.2018.09.017

[ref7] Dymek M. (2017). Expanding the Magic Circle–Gamification as a Marketplace Icon. Consum. Mark. Cult. 21, 590–602. doi: 10.1080/10253866.2017.1361153

[ref8] Garay-Rondero C. L. Rodríguez Calvo E. Z. Salinas-Navarro D. E. (2019). Experiential learning at lean-thinking-learning space. Int. J. Interact. Design Manufact. 13, 1129–1144. doi: 10.1007/s12008-019-00578-3

[ref9] Gou Z. Khoshbakht M. Mahdoudi B. (2018). The impact of outdoor views on students’ seat preference in learning environments. Buildings 8:96. doi: 10.3390/buildings8080096

[ref10] Griesinger D. H. (2018). Preference, localization, attention, and the limit of localization distance (LLD). J. Acoust. Soc. Am. 143:1932. doi: 10.1121/1.5036317

[ref11] Grzegorczyk G. (2019). The learning space in tutoring: how learning happens and/or does not happen. Chin. Semiot. Stud. 15:589–626. doi: 10.1515/css-2019-0031

[ref12] Haghighi M. M. Jusan M. B. M. (2015). The impact of classroom settings on students’ seat-selection and academic performance. Indoor Built Environ. 24, 280–288. doi: 10.1177/1420326X13509394

[ref13] Hemyari C. Zomorodian K. Ahrari I. Tavana S. Parva M. Pakshir K. (2012). The mutual impact of personality traits on seating preference and educational achievement. Eur. J. Psychol. Educ. 28, 863–877. doi: 10.1007/s10212-012-0144-3 PMC3355379 22611329

[ref14] Hong S. C. Lee J. (2017). Who is sitting next to you? Peer effects inside the classroom. Quant. Econ. 8, 239–275. doi: 10.3982/QE434

[ref26] Jarvis L. (2019). Narrative as virtual reality 2: revisiting immersion and interactivity in literature and electronic media. International Journal of Performance Arts and Digital Media 15, 1–2. doi: 10.1080/14794713.2019.1637100 30918619 PMC6417460

[ref15] Kalinowski S. Taper M. (2007). The effect of seat location on exam grades and student perceptions in an introductory biology class. J. Coll. Sci. Teach. January/February, 54–57.

[ref16] Kaya S. Ağaoğlu E. (2013). Opinions of instructors related to the physical layout dimension of virtual classroom management. Literacy Inform. Comput. Educ. J. 2, 1342–1350. doi: 10.20533/licej.2040.2589.2013.0178

[ref17] Lee K. S. Kim H. J. Kang J. (2019). From uniformity to sustainable diversity: exploring the design attributes of renovating standardized classrooms in Korea. Sustainability 11:5669. doi: 10.3390/su11205669

[ref18] Li Z. Li P. Zhou N. Liu Z. (2018). The research of embodied cognition learning environment design: characteristic, element, application and trend. J. Dist. Educ. 36, 81–90. doi: 10.15881/j.cnki.cn33-1304/g4.2018.05.011

[ref19] Liu Q.-T. Lu G.-Q. Wu L.-J. Deng W. (2021). Research on the correlation between location preference and learning motivation in smart classroom—take round table and seedling layouts as examples. Mod. Educ. Technol. 31, 67–75. doi: 10.3969/j.issn.1009-8097.2021.08.008

[ref20] Marx A. Fuhrer U. Hartig T. (1999). Effects of classroom seating arrangements on Children’s question-asking. Learn. Environ. Res. 2, 249–263. doi: 10.1023/A:1009901922191

[ref21] Mishra I. (2019). Gamification as a self-direct approach to attain instruction concepts in Indian school system. Open J. Soc. Sci. 7, 227–232. doi: 10.4236/jss.2019.75019

[ref22] Paas F. Merriënboer J. J. G. V. (2020). Cognitive-load theory: methods to manage working memory load in the learning of complex tasks. Curr. Dir. Psychol. Sci. 29, 394–398. doi: 10.1177/0963721420922183

[ref23] Parker T. Hoopes O. Eggett D. (2011). The effect of seat location and movement or permanence on student-initiated participation. Coll. Teach. 59, 79–84. doi: 10.1080/87567555.2010.538766

[ref24] Perkins K. K. Wieman C. E. (2005). The surprising impact of seat location on student performance. Phys. Teach. 43, 30–33. doi: 10.1119/1.1845987

[ref25] Rae K. Sands J. (2013). Using classroom layout to help reduce students’ apprehension and increase communication. Acc. Educ. 22, 489–491. doi: 10.1080/09639284.2013.835534

[ref27] Said I. Sahimi N. N. Rahman P. Z. M. A. (2015). Revealing young children and teachers behaviour through active participation in deciding classroom layout. Procedia. Soc. Behav. Sci. 168, 22–29. doi: 10.1016/j.sbspro.2014.10.206

[ref28] Seet H. A. A. Tan E. Rajalingam P. (2022). Effect of seating arrangement on class engagement in team-based learning: a quasi-experimental study. Med. Sci. Educ. 32, 229–237. doi: 10.1007/s40670-021-01469-7, PMID: 35154899 PMC8814094

[ref29] Shi X. Wang J. (2018). The effect of classroom setting on learning efficiency. Int. J. Sci. 5, 257–264.

[ref30] Stansbury J. A. Earnest D. R. (2016). Meaningful gamification in an industrial/organizational psychology course. Teach. Psychol. 44, 38–45. doi: 10.1177/0098628316677645

[ref31] Yang Y. Zhang J. (2021). The design framework of embodied learning activity in immersive virtual-reality fusion environment. Modern Dist. Educ. Res. 33, 63–73. doi: 10.3969/j.issn.1009-5195.2021.04.007

[ref32] Yin Y. Tang J. Wang S. (2021). Development of interactive network of adult education based on Bandura’s social learning theory. China Adult Educ. 20, 67–70.

[ref33] Zhang R. Zou D. Cheng G. Xie H. Wang F. L. Au O. T. S. (2021). Target languages, types of activities, engagement, and effectiveness of extramural language learning. PLoS One 16:e0253431. doi: 10.1371/journal.pone.0253431, PMID: 34181684 PMC8238195

[ref34] Zomorodian K. Parva M. Ahrari I. Tavana S. Hemyari C. Pakshir K. (2012). The effect of seating preferences of the medical students on educational achievement. Med. Educ. Online 17:10448. doi: 10.3402/meo.v17i0.10448, PMID: 22611329 PMC3355379

